# Activation of Midbrain and Ventral Striatal Regions Implicates Salience Processing during a Modified Beads Task

**DOI:** 10.1371/journal.pone.0058536

**Published:** 2013-03-06

**Authors:** Christine Esslinger, Urs Braun, Frederike Schirmbeck, Andreia Santos, Andreas Meyer-Lindenberg, Mathias Zink, Peter Kirsch

**Affiliations:** 1 Department of Psychiatry and Psychotherapy, Central Institute of Mental Health, Medical Faculty Mannheim, University of Heidelberg, Mannheim, Germany; 2 Department of Clinical Psychology, Central Institute of Mental Health, Medical Faculty Mannheim, University of Heidelberg, Mannheim, Germany; The University of Melbourne, Australia

## Abstract

**Introduction:**

Metacognition, i.e. critically reflecting on and monitoring one’s own reasoning, has been linked behaviorally to the emergence of delusions and is a focus of cognitive therapy in patients with schizophrenia. However, little is known about the neural processing underlying metacognitive function. To address this issue, we studied brain activity during a modified beads task which has been used to measure a “Jumping to Conclusions” (JTC) bias in schizophrenia patients.

**Methods:**

We used functional magnetic resonance imaging to identify neural systems active in twenty-five healthy subjects when solving a modified version of the “beads task”, which requires a probabilistic decision after a variable amount of data has been requested by the participants. We assessed brain activation over the duration of a trial and at the time point of decision making.

**Results:**

Analysis of activation during the whole process of probabilistic reasoning showed an extended network including the prefronto-parietal executive functioning network as well as medial parieto-occipital regions. During the decision process alone, activity in midbrain and ventral striatum was detected, as well as in thalamus, medial occipital cortex and anterior insula.

**Conclusions:**

Our data show that probabilistic reasoning shares neural substrates with executive functions. In addition, our finding that brain regions commonly associated with salience processing are active during probabilistic reasoning identifies a candidate mechanism that could underlie the behavioral link between dopamine-dependent aberrant salience and JTC in schizophrenia. Further studies with delusional schizophrenia patients will have to be performed to substantiate this link.

## Introduction

Metacognition, or “thinking about one's thinking”, comprises cognitive processes that monitor and control the subject’s own cognition [1]. In practice, metacognitive capacities involve the ability to select appropriate responses, to appraise and weigh information effectively and to cope with cognitive limitations. Patients with schizophrenia show deficits in several metacognitive abilities, which correlate with psychotic symptoms [2]–[4], and limited psychosocial function [5], [6]. Furthermore, in the last years, several aspects of metacognition have been implemented into therapies for schizophrenia [7]. In this context, a specific probabilistic reasoning bias known as the “Jumping to Conclusions” (JTC) bias has been associated to deficits in metacognition. This bias is characterized by the tendency to make hasty decisions and to rapidly accept beliefs, even when there is limited evidence supporting it [8]–[10]. It has been argued that schizophrenia patients, due to their difficulties to recognize themselves as agents during decisions, show this particular bias [11], [12] which might be associated with delusion formation. In the context of delusional disorders, this bias is investigated using versions of the so called “beads task”, were subjects have to guess from which of two jars or urns, containing beads of different ratios of colors, a sequence of beads are drawn [10].Buck and colleagues could very recently demonstrate that in patients from the schizophrenia spectrum, a reduced number of beads requested before concluding was associated with a lower level of subjective mastery, even after controlling for other cognitive factors like memory and executive functioning [11]. The authors interpret this finding as reflecting the important role of metacognition for the JTC bias in schizophrenia. The neural structures underlying this task has been evaluated in two imaging studies so far [13], [14]. The first study [13] reported an involvement of mainly cerebellar, parietal and occipital regions during probabilistic reasoning. However, this study involved only eight healthy subjects and applied a block design, which might not have been sensitive enough to differentiate higher metacognitive processes. The authors further used a fixed effect model, precluding generalization of the results. The second study [14] used a modified version of the beads task in the context of reward-related decision making. In this case subjects were able to win money if they chose the correct color and feedback was given after each decision. Behavioral data were investigated by comparing decision making behavior of the participant with that of an “ideal observer” as defined by a Bayesian model. Interestingly, under this reward condition, participants used less draws until decision than predicted by the model which could be described, although not mentioned by the authors, as hasty decision making or JTC bias. Brain imaging data were analyzed using an event related design comparing brain activation during decision with that during preceding draws. This analysis revealed a network comprising parietal, insular, anterior cingulate and striatal regions being more activated during the decision than during preceding draws. However, since jar choices were associated with reward feedback, the activation during decision making cannot be distinguished from reward anticipation processes activating a comparable network [15], [16]. Interestingly, when looking for increased activation during draws compared to jar choices, prefrontal (Brodmann Area 6, 8) areas were found. In addition dorsolateral prefrontal activation during decision making was positively correlated with the number of draws implicating more activation in those participants showing less hasty decision making behavior.

In the present study, we designed an fMRI-task on probabilistic reasoning according to the JTC paradigm to unravel its underlying neural networks. We tried to disentangle transient and sustained processes involved in probabilistic reasoning. Therefore we analyzed the task with a mixed model using a block design approach to identify neural networks that are related to the processing and maintaining of information before the participants draw their final conclusion, and applying additional event-related regressors to identify activity during the final evaluation of gathered data that leads to the decision.

It has been proposed that stimuli of the beads task elicit a salience signal [17], [18] Salience is meant as the feature of a particular stimulus in the environment that attaches attention, in doing so interrupts other cognitive foci and that potentially provides information to guide adaptive behavior. It has been proposed that such a reallocation of cognitive resources is driven by the ventral striatum and its dopaminergic inputs from midbrain regions [19]. In line with the information integration theory of probabilistic reasoning [20], as well as with the idea of a salience signal being elicited by stimuli in the beads task [17], [18], we hypothesize that regions related to salience processing, i.e. the ventral striatum and midbrain areas including the ventral tegmental area, should be activated during the task. This activation was expected to be most prominent during the last critical moments of reasoning just before arriving at a decision.

## Methods

### 1. Ethics Statement

After receiving written and oral instructions of the procedures, participants gave informed written consent. The study was approved by the local ethic committee of the Medical Faculty Mannheim of the University of Heidelberg (AZ 2009-296N-MA) and performed in accordance with the Declaration of Helsinki.

### 2. Participants

A group of 26 healthy volunteers (13 women, mean age: 28; range: 21–41 years; all right handed) participated in the study. Participants with neurological or psychiatric illness as well as history of substance dependence except nicotine were excluded. Data of one subject had to be excluded from analysis because of methodological reasons (see below).

### 3. Experimental Design

We designed a modified beads task adapted for use in functional imaging. Since beads tasks has been used to measure JTC bias in schizophrenia patients, we call it the “JTC task” although it has to be mentioned that the task does not measure JTC per se but allows to identify a JTC bias if present. The task was derived from the classical beads task [10] and included a more comprehensive, lifelike scenario which provides similar results as the original beads task [21], [22]. Subjects viewed fish of two colors jumping and had to decide from which of two lakes, containing fixed ratios (80/20% or 20/80%) of each type of fish, they were coming. Explicit information on the fixed ratios was provided to the participants. They could choose how many fish they wanted to see jumping out of the lake before they decided by pressing the key on the left/right side attributed to “yes” or “no” (fixed positions) with their right index or middle finger. Subjects were told they could view as many fish as they wanted without any pressure on speed or accuracy, just referring to the subjective level of confidence. For methodological reasons, however, the number of fish per block was restricted to ten. As the technique of functional MRI depends on repeated measures, the single JTC task was repeated eight times (eight task blocks, interleaved with eight control blocks, see below). During the task blocks, indicators at the side of the screen showed the colors of all previous fish [23], [24]. After presentation of each fish, subjects had to decide whether they wanted to see another fish or not. Once they decided they had seen enough fish or after the tenth fish they had to decide from which lake they came and then to rate on a four-point scale how confident they were about their decision (1 = a little uncertain, 2 = fairly certain, 3 = very certain, 4 = totally certain). To avoid stereotypical responses to all trials, the sequence in which the fish were presented changed in a preassigned fashion (see Table 1). JTC blocks were interleaved with a control condition requiring comparable visual and motor demands. In this task, a similar presentation of fish in two different colors was used but the task was only to indicate its color by pressing one of two buttons with the right index or middle finger (choice reaction task). The sequence and timing of stimulus presentation during the blocks was as follows: Two pools with fixed ratios of two colors of fish (80%/20% and 20%/80%) were introduced for 10 sec. After a variable fixation time (0.6–3.3 sec), fish in a preassigned order of colors were presented for 2 sec. Then a screen with the question (JTC block: “another fish?”; control block: “Name the Color?”) was presented until the subjects reached their decision. Between stimuli, a fixation cross was presented for 0.6 to 3.3 sec. Variable fixation times ensured that stimulus onset asynchrony (SOA) remained relatively stable at about 10 sec (mean SOA JTC blocks: 9.9 sec, min 7.6 sec, max 12.2 sec; mean SOA control blocks: 10.0 sec, min 7.9 sec, max 11.8 sec). In the JTC block, once the subject pressed “no”, a screen indicating the two lakes appeared together with the question from which lake the fish came. After the decision, subjects had to rate how confident they were about their decision. In the control condition, order of stimulus presentation was the same as in the task block, but subjects only had to indicate the color of the fish and there was no decision and no rating stimulus. The timing of all questions, namely whether the subjects chose another fish or not, the decisions for one or the other lake, the confidence ratings and the questions regarding the color of the control stimuli were self-paced by the subjects, i.e. the questions were presented until the subject pressed a button. To control for block length, the unequal lengths of the experimental blocks due to the different numbers of fish drawn by different subjects and different reaction times were counterbalanced by the number of repetitions of control trials in the following control block. As a result, subjects who drew less fish in the experimental block were presented with more trials in the control block and vice versa. Subjects were not made aware of this manipulation in advance of the experiment, but a monetary incentive was introduced to half of the sample (see below) to make sure that premature decisions made in order to shorten the experiment did not influence the decision process. Altogether, each round consisting of one JTC block and one control block lasted 2.2 minutes, adding up to 17.5 minutes for the whole experiment. JTC blocks lasted on average 16.2 sec (2.1–43.4 sec) and control bocks on average 39.2 sec (9.4–49.5 sec).

**Table 1:** Sequence of the color of fish in each block.

block #										
**1**	T	T	T	**O**	T	T	T	T	**O**	T
**2**	T	T	**O**	T	T	T	T	**O**	T	T
**3**	**O**	T	T	T	T	**O**	T	T	T	**O**
**4**	T	T	T	**O**	T	T	T	T	**O**	T
**5**	T	T	T	T	**O**	T	T	T	**O**	T
**6**	T	**O**	T	T	T	T	**O**	T	T	T
**7**	T	T	T	**O**	T	T	T	T	**O**	T
**8**	**O**	T	T	T	T	**O**	T	T	T	**O**

T = color of the target lake, O = color of the other lake. To introduce variety, colors changed in each block.

**Figure 1:**
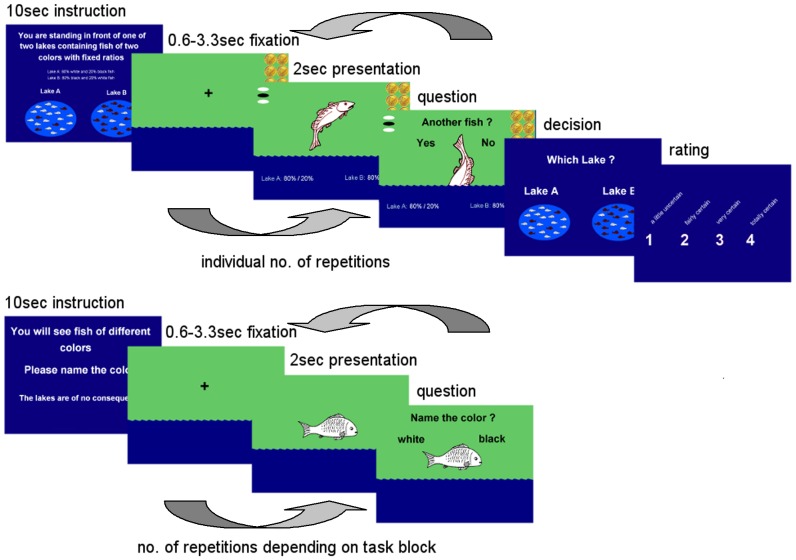
Jumping To Conclusions Paradigm, schematic of one of 8 task blocks: The schematic depicts the monetary incentive version where subjects saw the amount of money left for them to win in case they chose the right lake. For more detailed description of the task see the Methods section.

Half of the subjects were randomly assigned to a group that completed a monetary incentive version of the paradigm, in which subjects lost 20 cent from a stack of 2 euro for every additionally drawn fish, earning the rest of the money if they chose correctly in the end of a JTC test. No monetary manipulation was included in the control blocks. Neither group received feedback about the correctness of their decisions or the amount of money they won during the scanning session. Figure 1 displays the experimental design.

After scanning, data of one subject (female, 23 years, non monetary incentive version of the task) had to be excluded because she viewed all 10 fish during five of the eight blocks. No other subject decided to view more than nine fish in any block.

### 4. Data Acquisition and Data Analysis

Blood oxygen level-dependent (BOLD) fMRI was performed on a 3T Siemens Trio by using gradient echo, echo-planar imaging (28 axial slices, coplanar with a line through the anterior and posterior commissures; sequential order of acquisition; 4-mm thickness; 1-mm gap; TR/TE 2,000/28 ms; flip angle 80°; field of view 19.2 cm; matrix 64×64). Altogether, 526 scans were acquired and the first 4 volumes were discarded to account for saturation effects.

FMRI data was analyzed using SPM8 (www.fil.ion.ucl.ac.uk/spm/software/spm8/). Preprocessing steps included realignment to the first volume to correct for head motion, slice time correction to the middle slice, and normalization to a standard EPI template volume of the Montreal Neurological Institute (MNI) as provided with SPM8. Finally, a 9 mm full-width half-maximum Gaussian filter was applied to smooth the images.

All first level-analyses were performed using the general linear model approach of SPM. Task regressors were folded with SPM's canonical difference of gammas HRF. Prior to analysis, regressors and data were high pass filtered at 128 s and an autocorrelation model AR(1) was used. Data were analyzed in mixed or hybrid design fashion combining a block design and an event related design within one model [25]. Blocks were defined as starting with the first appearance of a fish stimulus and ending with the last decision about a stimulus in each condition (JTC blocks and control blocks). To further specify the decision process itself, event-related regressors were added to the model. One regressor contained stimulus onsets of all but the last fish and one regressor contained stimulus onsets of all eight last fish of the JTC blocks. Stimulus onsets in the control condition were modeled as regressors of no interest as were the onsets of decision making and confidence ratings. Reaction times to all stimuli were modeled as durations of the events.

Contrast images of task blocks minus control blocks were entered into a second level random effects one-sample T-test to identify general activation associated with the task. Contrast images of all last fish minus all preceding fish were then entered into the same type of second level analysis to identify activation associated with the decision.

In all first-level models, movement parameters from the realignement step of data preprocessing were included as covariates.

To test for the influence of motivation, second level random effects two sample t-tests between the classical and the monetary incentive version were performed with both the block design and the event related first level contrast images. Correlation of brain activation with behavioral measures was studied using random effects second level correlation analyses with the number of fish needed to come to a decision (draws to decision, DTD) and confidence ratings.

**Figure 2:**
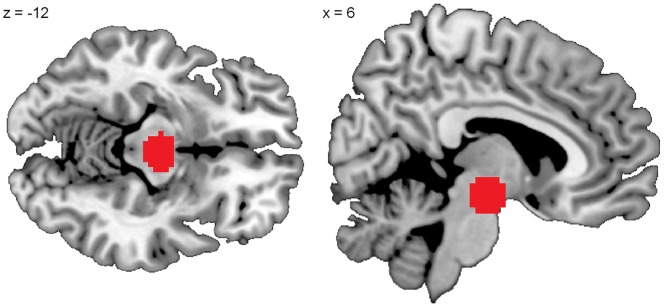
Mask for the ventral tegmental area (VTA): The region of interest was drawn manually on MRI-based anatomy using an anatomical atlas (Duvernoy HM (1995)).

For statistical inference, a threshold of p<0.05 with FWE (family wise error) correction for multiple testing across all voxels of the brain was applied. To specifically study activation in the ventral striatum (VS) and ventral tegmental area (VTA), region of interest (ROI) analyses within these regions were performed for all contrasts of interest. Statistical threshold for ROI analyses was p<.05, FWE corrected within a combined mask of VTA and bilateral VS. For VS we used masks from the Harvard-Oxford atlas comprising the right and the left nucleus accumbens, thresholded at a probability of 50% (distributed with the FSL software package; http://fsl.fmrib.ox.ac.uk/fsl/). For the VTA, a region of interest was drawn on MRI-based anatomy of the VTA region using an anatomical atlas [26], see Figure 2. Behavioral data were analyzed using SPSS version 16 (SPSS, Inc., Chicago, Illinois, USA). DTD and confidence ratings were analyzed using two-way ANOVA with the factors task version (classical versus monetary incentive) and block number (1–8).

## Results

### 1. Behavioral Data

**Figure 3:**
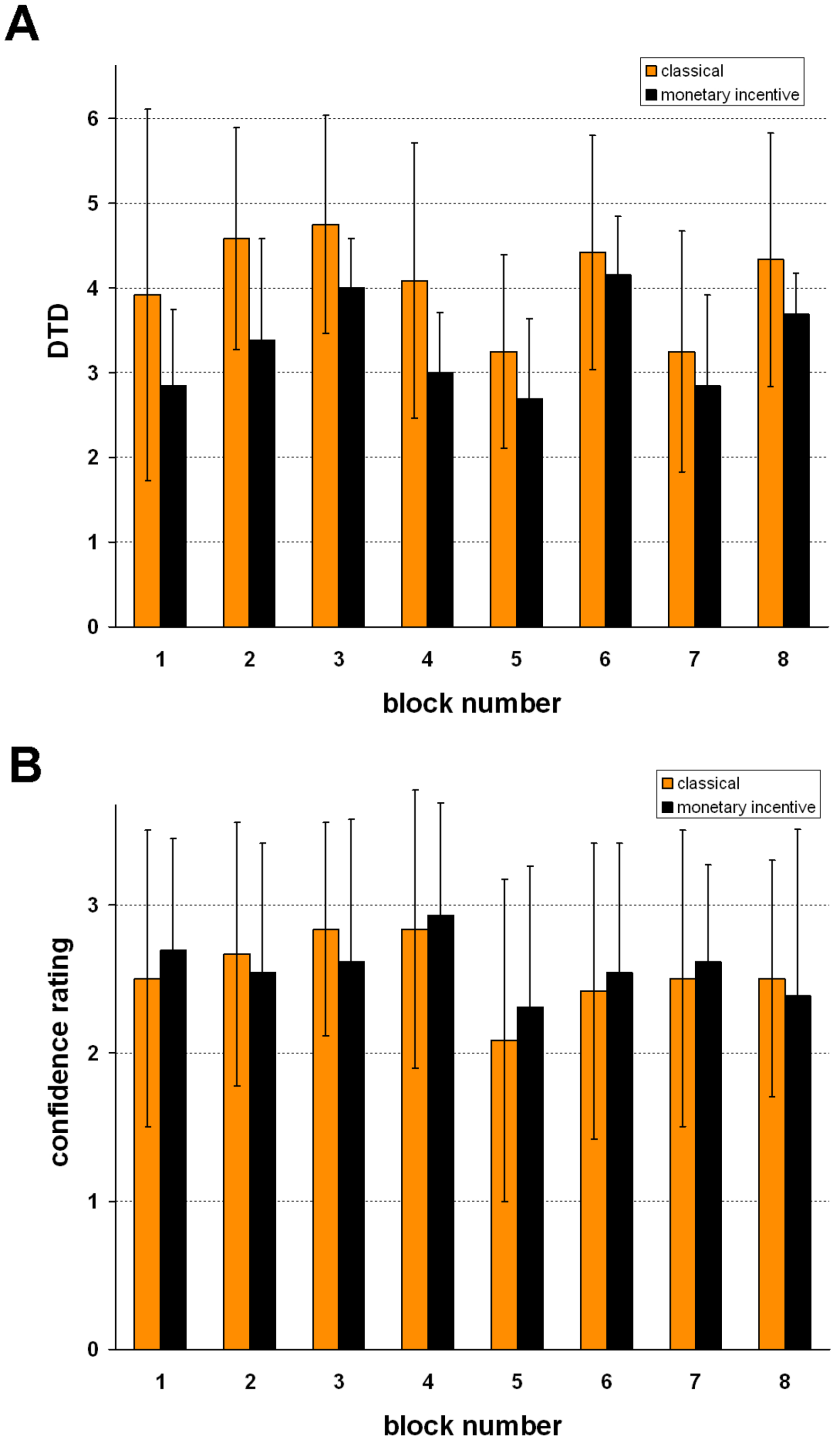
Means and standard deviations. **A:** Number of fish viewed per block before the decision was taken (draws to decision = DTD). **B:** Confidence ratings per block. (grey = classical version, black = monetary incentive version of the task).

Mean DTD was 3.69 (SD = 1.03) and depended significantly on the sequence in which fish were presented (main effect of block number: F(7,161) = 12.3, p = 3.7E-08). Mean DTD in each block is depicted separately for the classical and the monetary incentive version in Figure 3A.

In the monetary incentive version, in which subjects lost money with each draw, they tended to draw fewer fish (M = 3.33 in the monetary incentive vs. M = 4.07 in the classical version; main effect of task version: F(1/23) = 3.7, p = .069). The interaction between task version and block number was not found significant. Confidence ratings were on average 2.56 (fairly certain to very certain, SD = 0.63) and did not differ between the task versions (M = 2.58 in the monetary incentive vs. M = 2.54 in the classical version, F(1/23) = .002, p = .89). There was a tendency towards different ratings in the different blocks (one way ANOVA including block number as single within-subjects factor: F(7/161) = 2.16, p = .065) and no significant interaction between task version (classical/monetary incentive) and block number (see Figure 3B). DTD and confidence ratings did not correlate across blocks or across subjects.

### 2. Functional Imaging Data

#### 2.1. Activation during the modified beads task versus control condition

**Figure 4:**
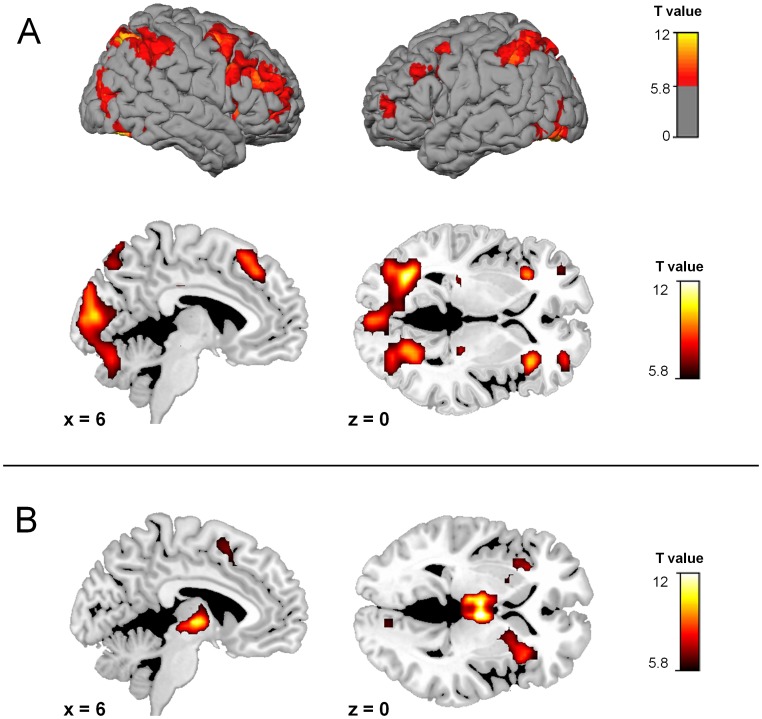
Brain activation. **A:** during Jumping to Conclusions (JTC) versus control blocks. **B:** at the time of last stimuli versus all preceding stimuli (event related regressors). Statistical significance family wise error (FWE) corrected: p<.05, cluster size> = 10 contiguous voxels).

Comparisons of the task blocks with the control blocks showed that during the task subjects activated the cerebellum, and superior parietal lobule stronger than during control blocks. In addition, we found significant activation in bilateral dorsolateral prefrontal gyrus, left insula, posterior cingulate, lingual gyrus, fusiform gyrus, supplementary motor area (SMA), bilateral thalamus and right brainstem. (Table 2, Figure 4A). Areas with greater activation in the control task than during the JTC task were right posterior insula (BA13), bilateral pre- and postcentral gyrus and right superior temporal gyrus (BA 22) (Table 3). ROI analyses of activation during the JTC versus the control condition revealed activation in the VTA (peak MNI coordinates [3 −25 −11], Tmax = 6.20, p = .00014, cluster size = 94). In the comparison classical against the monetary incentive version of the test, no significant activation differences could be detected in whole brain or in ROI analyses.

**Table 2:** Activation and deactivation during JTC versus control blocks: JTC>control.

hemisphere	anatomical region	BA	Tmax	Coordinates	k
**frontal lobe**					
right	middle frontalgyrus	BA 11/6	10.79	24 47 −11	1535^a^
	inferior frontalgyrus	BA 47	10.17	30 26 −5	1535^a^
	med. frontalgyrus/SMA	BA 6/8	9.57	6 32 43	236
left	inferior frontalgyrus	BA 47	10.43	−30 20 −5	91^b^
	precentralgyrus	BA 9	8.58	−42 5 34	132^c^
	middle frontalgyrus/SMA	BA 6/11/10/9	7.91	−45 8 52	132^c^
**insular cortex**					
left	insula	BA 13	7.58	−33 17 7	91^b^
**limbic lobe**					
right	cingulate gyrus	BA 23	7.47	6 −25 31	29
**temporal lobe**					
right	fusiform gyrus	BA 37	15.24	36 −58 −8	6658^d^
**parietal lobe**					
right	superior parietallobule	BA 7	15.62	27 −67 52	6658^d^
**occipital lobe**					
left	lingual gyrus	BA 19	14.44	−33 −64 −8	6658^d^
**Cerebellum**					
right	cerebellar tonsil		6.20	27 −40 −47	2
	inferior semi-lunarlobule		6.42	36 −64 −50	11
left	cerebellar tonsil		6.58	−21 −37 −50	6
	culmen		6.11	−6 −28 −17	5
**Subcortical**					
right	thalamus		7.76	27 −28 −2	26
left	thalamus		7.24	−27 −31 −2	19
**Brainstem**					
right	red nucleus		6.72	6 −25 −14	14

Significance threshold: p<.05, FWE (family wise error) corrected for the whole brain. BA = Brodmann area, Tmax = maximal t-value in the cluster, coordinates = MNI (Montreal Neurological Institute) coordinates of the peak voxel in the cluster. k = cluster-size, superscript letters indicate joint clusters.

**Table 3:** Activation and deactivation during JTC versus control blocks: control>JTC.

hemisphere	anatomical region	BA	Tmax	Coordinates	k
**frontal lobe**					
right	precentralgyrus/SMA	BA 6/4	7.62	27 −19 73	140^a^
left	precentralgyrus	BA 43	9.27	−54 −10 10	458
	superior frontalgyrus/SMA	BA 6	8.41	−18 −10 73	171^b^
	medial frontalgyrus/SMA	BA 6/11	6.95	0 −10 52	57
**parietal lobe**					
right	postcentralgyrus	BA 3/7	7.13	24 −34 73	140^a^
left	postcentralgyrus	BA 5/40/3	10.86	−24 −43 73	171^b^
	angular gyrus	BA 39	8.05	−45 −79 37	17
**limbic lobe**					
right	anterior cingulate	BA 32	8.18	3 23 −8	56^c^
	posterior cingulate	BA 31	6.64	3 −58 25	37
	cingulate gyrus	BA 31	5.95	18 −28 49	1
left	anterior cingulate	BA 24	7.99	−6 26 −5	56^c^
**insular cortex**					
right	insula	BA 13	9.72	42 −19 4	670^d^
**temporal lobe**					
right	superior temporalgyrus	BA 22	9.52	57 2 4	670^d^
	angular gyrus	BA 39	7.96	54 −73 25	7

Significance threshold: p<.05, FWE (family wise error) corrected for the whole brain. BA = Brodmann area, Tmax = maximal t-value in the cluster, coordinates = MNI (Montreal Neurological Institute) coordinates of the peak voxel in the cluster. k = cluster-size, superscript letters indicate joint clusters.

Correlation analyses revealed one cluster in the left middle temporal gyrus (BA22) that showed increased activation during the JTC task versus the control task with increased confidence rating (MNI coordinates [−63 −22 −16], Tmax = 6.25, p = .028, cluster size = 9) Activation in no other regions correlated positively or negatively with DTD or confidence ratings as assessed with whole brain or ROI analyses.

#### 2.2. Activation during the moment of decision making

Comparing all last fish vs. all preceding fish we found significantly increased activation during presentation of the last fish compared to the preceding ones in right ventrolateral prefrontal cortex, right parahippocampal gyrus, lingual gyrus, bilateral putamen, bilateral thalamus and bilateral insula and left brainstem (Table 4, Figure 4B). ROI analyses showed activation in the VTA ([6 −13 −5], Tmax = 10.5, p = 3.8E-8, cluster size = 129) and right VS ([12 8 −8], Tmax = 4.23, p = .01, cluster size = 4). No significant activation in grey matter in the opposite contrast: all previous>last stimulus was revealed in the whole brain or the ROI analyses.

**Table 4:** Activation during presentation of the last versus all preceding fish (event related regressors): last >preceding fish.

hemisphere	anatomical region	BA	Tmax	coordinates	k
**frontal lobe**					
right	medial frontalgyrus	BA 32/9	6.89	9 8 52	122^a^
	inferior frontalgyrus	BA 9	6.05	45 5 31	1
left	medial frontalgyrus	BA 32	7.58	−9 11 49	122^a^
**parietal lobe**					
right	postcentral gyrus	BA 2	6.75	42 −28 37	1
**limbic lobe**					
right	parahippocampal gyrus	BA 19	6.48	33 −49 −8	4
**insular cortex**					
right	Insula	BA 13	9.24	33 17 −5	277^b^
left	insula	BA 13	7.26	−36 14 −5	147^c^
**occipital lobe**					
right	lingual gyrus	BA 18	6.43	12 −82 −2	18
**Subcortical**					
right	Thalamus		14.61	6 −13 −2	312^d^
	Putamen		7.56	18 5 −8	277^b^
left	Thalamus		12.79	−3 −13 −2	312^d^
	Claustrum		8.71	−27 23 4	147^c^
	Putamen		7.53	−18 8 10	147^c^
**Brainstem**					
left	red nucleus		11.11	−6 −22 −5	312^d^

Significance threshold: p<.05, FWE (family wise error) corrected for the whole brain. BA = Brodmann area, Tmax = maximal t-value in the cluster, coordinates = MNI (Montreal Neurological Institute) coordinates of the peak voxel in the cluster. k = cluster-size, superscript letters indicate joint clusters.

When testing this contrast in the monetary incentive versus the standard version, neither whole brain analysis nor ROI analysis revealed any significant differences between conditions.

To determine whether activation during presentation of the last versus all previous fish depended on the number of fish drawn (DTD) or on confidence of the subsequent decisions, we performed voxelwise correlation analyses of activation in the contrast last versus all previous fish with mean DTD and mean confidence ratings over the 8 blocks. None of the analyses showed correlation between brain activation and DTD or confidence ratings at an FWE corrected significance level for the whole brain or within the ROIs.

## Discussion

We investigated the neural networks involved in decision making under uncertainty during the so-called “Jumping to Conclusions” (JTC) paradigm. Functional imaging showed an extended executive cognition network of sustained activation during reasoning, while ventral striatal regions, which have been associated with saliency [27]–[29], were activated more strongly during the final stage of the particular decision as compared to its initiation.

In a behavioral perspective, participants asked for a comparable amount of evidence before coming to a conclusion to what has been observed in healthy subjects in previous studies [10], [21], [30]. As expected, DTD depended strongly on the order of stimulus presentation. There was a trend towards fewer DTD in the monetary incentive version possibly due to the fact that subjects had to “pay” for each additional fish drawn, but the difference to subjects performing the classical version of the test was not significant. Together with the absence of group differences in confidence ratings, this pattern of performance supports the assumption that subjects did not “jump to conclusions” out of lack of motivation and to shorten the experiment. Furthermore, it may indicate that incentive motivation contributes only marginally to the activation patterns.

The task-specific recruitment of neural structures involved an extended network associated with executive functioning: right ventrolateral and bilateral dorsolateral prefrontal cortex, superior and inferior parietal lobule and precuneus, premotor regions and pre-SMA [31] Although it cannot be excluded that the network activated also reflects other processes that differ between the beads task and the control task but is independent of the decision making process (e.g. increased visual processing of additional information, increased attentional demands or reading of the lake proportions etc. ), it is interesting that these regions were also found to be involved in the rewarded beads task used by Furl and Averbek [14]. However some of these regions (ventrolateral prefrontal cortex, precuneus, pre–SMA were found to be more activated during draws than during decision in this study while others were found to be more activated during decision (posterior regions, insula). In addition, we confirmed activation in regions previously described by Blackwood and colleagues [13], medial occipital cortex and cerebellum. This previous study might have masked activation in the executive functioning network, in particular the DLPFC, because of differences in the control condition, where Blackwood and colleagues required participants to monitor the frequencies of stimuli and remember them during the whole block. This introduced a working memory component not present in our control condition which was to reach a decision under certainty (color of each stimulus) after each stimulus separately. Even if all information about previously collected data is visible at all times during the reasoning process, our data therefore support executive functions and working memory as a core component of this specific probabilistic reasoning test. The fact that none of these main regions of the executive functioning network were differentially activated at the time of the last versus all other stimuli indicates that executive control is required throughout the process of reaching a decision. Our findings show that differentiating between sustained and transient processes during the beads reveals an important network involved in the problem solving part of the task which cannot be clearly assigned to a particular process when using a block or event related design exclusively.

When analyzing the classical and the monetary incentive versions of the task separately, we did not find activation differences in the block design analysis. This corresponds to the absence of significant differences at the behavioral level, and supports the view that the experimental situation and the task by itself is motivating enough, at least for healthy controls, and subjects did not prematurely discontinue the trials to shorten the experiment. Usually, we would have expected increased activation in reward related brain regions such as ventral striatum and orbitofrontal cortex in the monetary incentive versus the nonincentive version of the task [14]. The reason why we did not see such activation might be that participants did not receive feedback regarding the correctness of their decisions and therefore did not experience anticipation of reward during the presentation of task stimuli. In addition, differences might be covered by the reduced group size. Therefore it might have been preferable to expose all subjects to all conditions in a fully factorized design increasing the chance to detect effects of this manipulation.

In our second analysis, aimed at investigating activation at the moment of decision making, we found activation in structures linked to salience and dopaminergic neurotransmission nicely replicating the findings reported by Furls and Averbek using their rewarded version of the beads task [14]. Regions that were more activated during the last stimulus that led to a decision compared to all preceding stimuli were bilateral striatum and midbrain including the ventral tegmental area, brain areas that are reliably activated in salience processing [27], [29], [32]–[35]. This finding is in line with our hypothesis that salience processing might play a prominent role in decisions under uncertainty. The ventral striatal regions found here, were not only associated with salience but also with other processes like reward anticipation 1(e.g. [15], [16]). However, since we could not find differences between our rewarded and our unrewarded version in these regions, we conclude that the increased activation to the last fish is mainly driven by the acquired salience of this stimulus rather than its rewarding value. This conclusion is also in accordance with studies demonstrating the modulation of ventral striatal activation by salience, even in the context of monetary reward [33] or aversive context conditioning [28].

Additionally, we found right VLPFC, pre-SMA, bilateral insula, medial occipital regions and bilateral thalamus to be transiently active. Interestingly and further supporting our conclusion of the role of saliency in our task, anterior insula is also implicated in the salience network [36], and seems to play a role in encoding uncertainty [37], [38]. Thalamic, especially medial regions (dorsomedial nucleus) have tight connections with prefrontal cortex and are relevant for the dopaminergic control of processing of sensory information [39].

Taken together, the increased activation in brain regions known to support dopaminergic and salience related cognitive functions, we speculate that the last fish before decision might constitute a highly salient signal with a marked subjective importance attributed to the provided color information, which would then ultimately trigger the a response to stop gathering evidence an come to a conclusion. In contrast, the block design analysis would capture activation related to the preparation of the decision during the whole block by a more cognitive process that recruits the executive functions network. The decision for on or the other lake would then not exclusively be based on ongoing cognitive calculations of probabilities as represented by prefrontal activation, but would at least partly be driven by a salience signal from the ventral striatum preceding the actual decision.

Menon and colleagues [17] failed to see the typical behavioral JTC response pattern in schizophrenia patients in a version of the task where they showed a memory aid and postulated a possible influence of memory load on the distortion of the stimulus salience, although other authors have seen JTC bias even with no memory load [23], [24]. Here, he we found salience regions clearly activated at decisions without memory load. We found no significant correlations between brain activation and probabilistic reasoning styles, assessed by numbers of stimuli viewed before reaching a decision (DTD) and confidence ratings. Again this might be because we studied only healthy subjects without a broad enough range of behavioral differences.

When comparing the two versions of the task in the event-related analysis, we found more activation in the VTA in the classical version. Because at the moment of decision, the participants did not receive feedback about whether they actually gained or lost money, it cannot be excluded that this finding reflects relatively reduced importance of the salience system at the moment of decision in the monetary incentive version because of a sustained elevated level of reward-related activation of this system during the whole block of the JTC condition.

Our data show that, at least in healthy individuals, the JTC test activates regions implicated in salience processing and might provide a neural mechanism that could form a link between aberrant salience processing and the formation of metacognitive biases such as the JTC bias [17], [18]. To speculate further, aberrant and untimely spiking dopaminergic neurons might be a neurobiological correlate of false attribution of salience to stimuli relevant for decisions. In schizophrenia, because the dopamine system is dysregulated [40], this process might be chaotically upregulated which could be inferred from the altered activation of the dopaminergic midbrain and striatum during reward learning found in psychosis [41], and which might explain both the tendency for JTC and the marked heterogeneity of patients’ performance in the JTC test.

Our data leave the question open whether the metacognitive bias in JTC is a cognitive link from aberrant salience processing in schizophrenia to delusion formation [42]–[44]. Behavioral evidence showing higher rates of JTC bias in delusional schizophrenia patients than in patients without delusions [23], [45] indicate that delusions and the JTC bias share variance. In addition, since our block design analysis found a tonic activation of prefrontal cortex during the decision process, it is possible that impairments in executive functioning and working memory, commonly found in schizophrenia, contribute to the JTC bias in this disorder, either independently or interacting with aberrant salience processing. Behaviorally, it has been shown that the JTC bias in schizophrenia is related to, but not completely dependent on, executive functioning, in particular mental flexibility [21], [46], [47]. To test these relationships, additional studies with schizophrenia patients before and during antipsychotic treatment will have to be performed. The proposed mechanism might be specifically important in at-risk-mental states (ARMS), when first delusional symptoms are reported. The JTC neuroimaging paradigm and the differentiated analysis reported here should be an appropriate experimental approach to further our knowledge about the neurobiological underpinnings of this specific metacognitive deficit in schizophrenia.
